# Mechanisms for the temporal regulation of substrate ubiquitination by the anaphase-promoting complex/cyclosome

**DOI:** 10.1186/s13008-019-0057-5

**Published:** 2019-12-23

**Authors:** Shivangee Bansal, Swati Tiwari

**Affiliations:** School of Biotechnology, Jawaharlal Nehru University, New Delhi, 110067 India

**Keywords:** Anaphase-promoting complex, APC/C, Cell cycle, Substrate ordering, Ubiquitination

## Abstract

The anaphase-promoting complex/cyclosome (APC/C) is a multi-subunit, multifunctional ubiquitin ligase that controls the temporal degradation of numerous cell cycle regulatory proteins to direct the unidirectional cell cycle phases. Several different mechanisms contribute to ensure the correct order of substrate modification by the APC/C complex. Recent advances in biochemical, biophysical and structural studies of APC/C have provided a deep mechanistic insight into the working of this complex ubiquitin ligase. This complex displays remarkable conformational flexibility in response to various binding partners and post-translational modifications, which together regulate substrate selection and catalysis of APC/C. Apart from this, various features and modifications of the substrates also influence their recognition and affinity to APC/C complex. Ultimately, temporal degradation of substrates depends on the kind of ubiquitin modification received, the processivity of APC/C, and other extrinsic mechanisms. This review discusses our current understanding of various intrinsic and extrinsic mechanisms responsible for ‘substrate ordering’ by the APC/C complex.

## Background

Eukaryotic cell cycle is a unidirectional, ordered event that is controlled by a large number of regulatory proteins. Ubiquitination and proteasomes play crucial roles in determining the stability of numerous cell cycle regulatory proteins, and in ensuring the directionality of the cycle. Ubiquitination is catalyzed in three sequential steps. It starts with ubiquitin activating enzyme (E1) forming a thioester bond at its conserved cysteine with C-terminal glycine residue of ubiquitin (UB), in a reaction that utilizes ATP. Activated ubiquitin then forms a thioester bond with the active site cysteine residue in the ubiquitin conjugating enzymes (E2s). Finally, ubiquitin ligase enzymes (E3s) facilitate transfer of UB from E2 to target substrates. Modified substrates are either degraded by the proteasomes or subjected to regulatory mechanisms depending on the length and kind of UB chain [1]. The anaphase-promoting complex/cyclosome (APC/C) and SKP/Cullin/F-box containing complex (SCF complex) are two highly conserved E3s belonging to the multi-subunit cullin-RING ligase (CRL) family, which regulate the turnover of a large number of cell cycle regulatory proteins besides having non-cell cycle substrates. SCF is active throughout the cell cycle, requires phosphorylation of its substrates for their recognition, and plays an important role in the G1/S and G2/M transitions [2]. APC/C activity is manifested upon phosphorylation of its subunits and is assumed to be restricted to late M and G1 phases, promoting degradation of distinct cell cycle regulators. But recent studies suggest that it is also important for DNA damage checkpoint arrest in G2 as well [3–5].

The large size and complex structure of APC/C have been a challenge for a detailed understanding of this multi-subunit E3 enzyme till recently. Structural studies, using cryo-electron microscopy, X-ray crystallography and NMR, along with many enzymatic studies have together provided an almost atomic level model of the APC/C. These studies have provided a detailed understanding of how this complex recognizes and modifies a large number of substrates in a temporally regulated manner depending on the cell cycle phase. Complex and multiple controls, that utilize many different mechanisms, contribute to achieve a spatio-temporal order of substrate modification by APC/C during the cell cycle [6, 7]. Although great advances have been made on the details of its activation, substrate selection and processivity, its complete understanding still remains elusive. Additionally, the APC/C complex has now been shown to have numerous other cell cycle independent functions. Several of these previously unknown functions of APC/C need to be coordinated with the cell cycle. Given the range of APC/C functions and substrates, the principles governing the substrate ordering are complex and varied. This review is focused on the current understanding of various mechanisms that temporally regulate susbstrate binding and ubiquitination by the APC/C.

## Main text

### Structural organization and conformational flexibility of APC/C

**Fig. 1:**
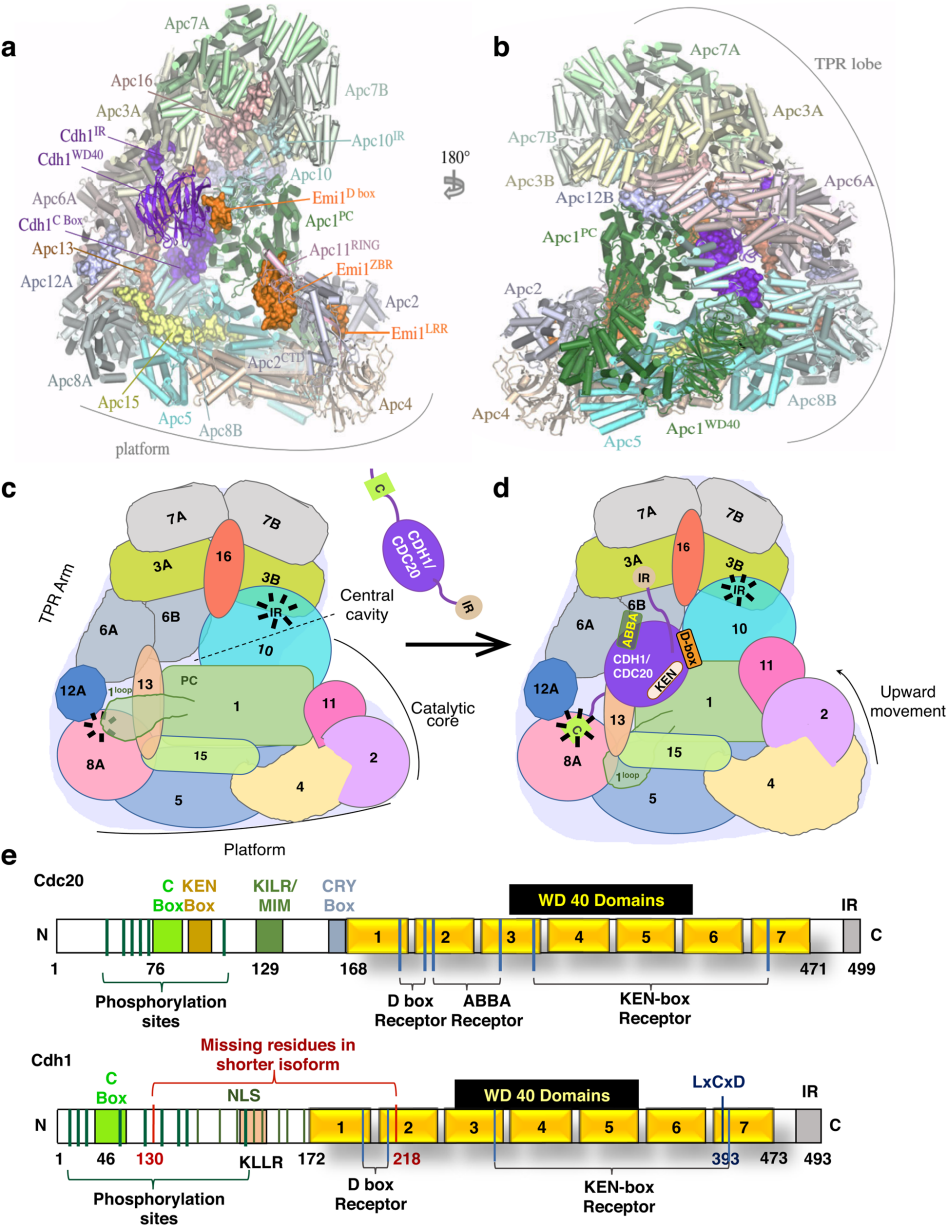
Structural organization of the APC/C. **a**, **b** EM construction of the APC/C^CDH1^ in complex with the inhibitory protein Emi1. Reproduced with permission from [9]. **c** Schematic of the Apo-APC/C. **d** active APC/C. Positions of IR tail binding sites and C-box binding sites are shown. Change in the mobility of the platform and repositioning of the catalytic core upon substrate binding is indicated. **e** Domain organization and modification sites of CDC20 (upper) and CDH1 (lower) with known CDK1 phosphorylation sites (dark green)

We briefly present here the structural organization of APC/C; for details, readers may consult other recent excellent reviews [8–11]. The APC/C is a very large complex of about ~ 1.2 Mda. The core complex, composed of 14 subunits in metazoa (13 in yeast), interchangeably associates with coactivator subunits [12]. Since most of the APC/C subunits are essential, building an initial topological map of APC/C required developing a yeast strain that could survive without APC/C activity. This strain provided the crucial first information about the topological arrangement of various subunits of the budding yeast APC/C. The overall shape of the complex was found to be triangular with tetratricopeptide (TPR) lobes forming a bowl-shaped structure above the platform (Fig. 1), thus resembling an ‘arc-lamp’ like architecture with a central cavity [13]. Later, a combination of crystallographic data of various subunits and subcomplexes, mass spectroscopy and advances in single particle cryo-EM, yielded data from yeast and vertebrate APC/C providing a near atomic-level description of the complex (Fig. 1a, b). Together, these studies have provided a deeper insight into the conformational flexibility of the complex and how it is linked to its functions [9, 14].

On the functional basis, the whole complex of APC/C is divided into four domains: catalytic core (APC11 containing RING H2 domain tightly associated with APC2 cullin subunit), scaffolding core (APC1, APC4, APC5, APC15), TPR subunits (APC6/CDC16, APC3/CDC27, APC8/CDC23), and coactivators CDC20/Fizzy (Drosophila) and CDH1/Fizzy related-1 (FZR1) [15, 16]. Based on its architecture, the complex is described to have a “Platform” made up of APC1, APC4, APC5, APC15, and the “Arc lamp” that has the scaffolding TPR subunits APC8/CDC23, APC6, APC3 (Fig. 1a, b). The N-terminal of these TPR subunits form a superhelix with a pseudo dyad symmetry, while their C-terminals fan out to the convex and concave curvatures of the complex. The asymmetric arrangement of the TPR subunits is stabilized by the non-TPR subunits, APC16, and APC13 passing through APC8/APC6 and APC3/APC7 interfaces. APC6 is stabilized by its interaction with APC12/CDC26.

The TPR subunits are suggested to provide a scaffold for the assembly of the complex and provide binding sites for substrates and regulatory subunits [16]. The scaffold anchors the catalytic and substrate recognition modules on opposite sides in a way that they face each other (Fig. 1c). The coactivators and co-receptor APC10/DOC1 are connected via their C-terminal Ile-Arg motifs (IR-tails) to the grooves in the C-terminal of homodimeric, APC3/CDC27 subunits in the TPR-lobe [17, 18]. The C-box domain of the coactivators engages with a groove in APC8/CDC23 that is structurally similar to the IR-tail binding grooves in APC3/CDC27. This arrangement allows the substrate binding WD40 domain of coactivators to be flexible in its position depending on the binding partners. The structural similarity of the C-box-binding region of APC8/CDC23 and IR-tail binding regions of APC3/CDC27 is important for the functional regulation of the APC/C by the mitotic checkpoint complex (MCC), which binds to the APC/C complex via interaction of the IR-tail of the CDC20 with the C-box of APC8/CDC23.

APC1 subunit of the platform provides an anchor to other APC subunits and its orientation controls the position and flexibility of the catalytic core of APC2 and APC11. Therefore, it is critical for regulating the APC/C ubiquitination activity. APC1 has eleven Proteasome-Cyclosome (PC) repeats at its C-terminal that interact with the TPR lobe and the coreceptor subunit, APC10/DOC1. The N-terminal WD40 domain of APC1 is necessary for promoting the binding affinity of the E2 UBE2C/UBCH10 [19]. APC1 also interacts with the N-terminal domain of the coactivator CDH1/FZR1 (Fig. 1d). As discussed later, APC1, in a phosphorylation dependent manner, also regulates the engagement of CDC20 to the complex.

The catalytic core is formed by APC11 and APC2. The RING domain of APC11, and the winged-helix B (WHB) domain of APC2, are connected via flexible linkers to the C-terminal domain of APC2 (Fig. 1a, b). The flexibility of the catalytic module is critical for APC/C functions and is influenced both by the orientation of the platform, as well as by direct interaction partners of APC11/APC2.

The platform and the scaffold form a central cavity. The coactivator and APC10/DOC1 are positioned at the top of the cavity with extensive interactions between APC1 and APC10/DOC1 (Fig. 1a–d). The catalytic core is at the front of the cavity and faces the coactivator module (Fig. 1a, b). Thus, both extensive and flexible contacts of the platform and substrate recognition module with the scaffold; and between the platform and the catalytic module, allow a remarkable conformational flexibility of APC/C that is controlled by various binding partners and post-translational modifications.

## APC/C functions

### **APC/C in cell cycle regulation**

APC/C tightly governs cell cycle progression by controlling metaphase to anaphase transition and mitotic exit. It also plays a pivotal role in governing the next cycle through the G1 phase and in regulating DNA damage response in G2 [4, 20]. To carry out these functions, the activity of APC/C is modulated by several coactivators [21]. These coactivators modulate APC/C activity by conformational changes in the APC/C complex organization [22, 23]. To a large extent, two coactivators CDC20/Fizzy or CDH1/FZR1, temporally modulate APC/C activity during mitosis. By associating with APC/C in different phases of the cell cycle, these coactivators can target different as well as overlapping substrates. Moreover, they oppose each other’s activities, which is important for the progression of the cell cycle. During meiosis, other coactivators belonging to CDC20 family participate in forming activated APC/C complex. In budding yeast, Meiotic fizzy related-1 (MFR1), Activator of meiotic APC1 (AMA1) are such coactivators of CDC20 family that are expressed to coordinate meiotic exit and cytokinesis [24, 25]. In mammals, APC/C^CDH1^ plays an important role during meiosis at the G2/M boundary and prometaphase I progression in females. APC/C function in meiosis has been discussed in several recent reviews [26, 27] and, thus, we will not discussed it further.

#### APC/C^CDC20^ mediated processes

Phosphorylation of APC/C subunits by Cyclin B/CDK1 complex triggers APC/C^CDC20^ activity [28] which is required to promote the metaphase to anaphase transition. Major substrates of APC/C^CDC20^ are NEK2A, Cyclin A, Cyclin B, and Securin. It is important to prevent the degradation of Cyclin B and Securin till all the sister chromatids are properly attached to the kinetochore. Therefore, APC/C^CDC20^ activity is kept in check until the spindle assembly checkpoint (SAC) is satisfied. Neverthless, APC/C^CDC20^ targets NEK2A and Cyclin A in prometaphase, immediately after the nuclear envelope breakdown, while the SAC checkpoint is active [29, 30]. Cyclin B is degraded at metaphase by APC/C^CDC20^, which reduces the kinase activity of CDK1, consequently triggering the anaphase progression. The metaphase to anaphase transition requires separation of the sister chromatids; this is catalyzed by Separase. Further, Separase is inhibited by Securin, which is an essential substrate of APC/C^CDC20^. At anaphase entry, APC/C^CDC20^ mediates polyubiquitination of Securin, leading to its proteasomal degradation. In turn, Separase is released and becomes active resulting in Cohesin cleavage [31]. Thus, Cyclin A and NEK2A are insensitive to SAC while other mitotic substrates are sensitive to it. Cyclin A is degraded early even if the SAC is defective or absent [32]. Similarly, CLB5 in budding yeast also gets degraded before Securin in the cells that do, or do not, have SAC [33]. Therefore, SAC independent mechanisms govern the timely degradation of these substrates.

#### APC/C^CDH1^ mediated processes

Degradation of mitotic cyclins, and resulting dephopshorylation of CDH1, allows it to interact with APC/C, leading to CDC20 ubiquitination and degradation. Other substrates like Polo-like kinase-1 (PLK1), Aurora kinase A, and Aurora kinase B are ubiquitinated for mitotic exit after they have performed their functions in the telophase and during cytokinesis [34–36]. CDH1 maintains the ubiquitination of Securin until the end of G1 phase and ensures low kinase activity throughout the G1 phase by degrading mitotic cyclins [37, 38]. Once these substrates have been degraded, autoubiquitination of the E2 enzyme UBE2C/UBCH10 is promoted in G1, leading to inactivation of APC/C^CDH1^ and stabilization of Cyclin A [39]. Premature entry into the S-phase is prevented by APC/C^CDH1^ by abolishing the regulators of replication such as ORC1, CDC6, and Geminin [40]. Additionally, by interacting with Retinoblastoma protein (pRB), APC/C^CDH1^ promotes the degradation of SKP2, which is the substrate recruiting subunit of the SCF complex. Degradation of SKP2 facilitates the accumulation of SCF substrates, like the cyclin-dependent kinase inhibitor (CKI) p27^KIP1^, and prevents untimely entry into the S-phase [41]. In contrast to the restricted set of substrates of APC/C^CDC20^, APC/C^CDH1^ has numerous substrates that have been identified to date.

### Cell cycle independent functions of APC/C

Recent studies on APC/C reveal that it has a much broader involvement in diverse cellular functions including developmental processes, differentiation, function of nervous system, genomic stability, tumor suppression, apoptosis, senescence, energy metabolism, and cell motility [42–48]. Interestingly, most of the non-cell cycle functions of APC/C are intimately coordinated with the cell cycle. But there is very limited understanding of how this coordination is achieved. A few examples of non-cell cycle substrates of APC/C whose degradation is likely to be coordinated with cell cycle are described below.

HOXC10, a member of HOX family of transcription factors, is present from arthropods to vertebrates. HOX family proteins are important for growth control along the embryonic body axis and are targeted by APC/C for degradation in early mitosis. The timing of degradation of HOXC10 coincides with that of Cyclin A, suggesting a link between APC/C function and development [49]. However, the mechanism that allows SAC-independent degradation of HOXC10 is not understood.

APC/C activity is coupled to many aspects of neural functions like axon growth, morphology, stem cells proliferation, and differentiation. CDH1 is highly expressed in mature neurons in the central nervous system and controls neural stem cells (NSCs) proliferation and differentiation into neurons [50]. In all trans-retinoic acid induced NSCs, APC/C^CDH1^ activity is up-regulated [50] and the Inhibitor of differentiation 2 (ID2), which is a substrate of APC/C^CDH1^, is down regulated [51]. During axonal morphogenesis in mammalian brain, nuclear APC/C^CDH1^ targets SnoN, a transcriptional corepressor of TGFβ signalling, and a potent promoter of axonal elongation in primary neurons [52]. APC/C^CDH1^ dependent degradation of SnoN and SKP2 in TGF-β signaling has been proposed to be coordinated through temporal management of the substrates [53]. TGF-β induced degradation of SKP2 by APC/C^CDH1^ withdraws cells from cell cycle progression and subsequently activates differentiation program [53].

APC/C^CDH1^ also regulates muscle differentiation by targeting the cell fate determining myogenic factor, MYF5 [54, 55] to maintain its basal level. Additionally, D-box dependent degradation of SKP2 by APC/C^CDH1^ allows elevated levels of p21^CIP1^ and p27^KIP1^ [56, 57], achieving cell cycle arrest. Thus, APC/C^CDH1^ modulates muscle differentiation by coordinating cell cycle progression with initiation of myogenic differentiation program.

Metabolic processes required for cell duplication also need to be coordinated with cell cycle progression. Glucose and glutamine provide raw materials for the synthesis of macromolecules required for cell division. 6-phosphofructo-2-kinase/fructose-2, 6-biphosphatase isoform 3 (PFKFB3) and Glutaminase-1 (GLS-1) are key enzymes involved in glycolysis and glutaminolysis, respectively. PFKFB3 generates fructose 2,6-bis-phosphate, an allosteric activator of 6-phosphofructo-1-kinase [58], while Glutaminase-1 converts glutamine to lactate during glutaminolysis. Both PFKFB3 and GLS-1 are substrates of APC/C^CDH1^ and their levels are coordinately regulated with cell proliferation and lactose generation [59, 60]. These studies suggest a unifying link between APC/C activity, metabolism and cell cycle [61].

MCL-1 is an anti-apoptotic protein regulated by APC/C^CDC20^ during cell cycle. When cells fail to resolve mitotic arrest, MCL-1 gets phosphorylated at critical sites by Cyclin B/CDK1, then gets recognized by APC/C^CDC20^ and ubiquitinated for degradation via proteasomal pathway [62]. This is suggestive of a temporal mechanism that can distinguish between normal and prolonged mitosis to control the degradation of substrates of mitosis and apoptosis.

The above examples point to the connection between cell cycle and other cellular activities of APC/C. All these substrates of APC/C are likely to be degraded in an orderly fashion to orchestrate the unidirectional cell cycle events, and simultaneously regulate other cellular functions. Most of what we understand about temporal regulation of APC/C activity and substrate ordering is derived from its functions in cell cycle. However, not much is understood about how APC/C discriminates among its cell cycle and non-cell cycle substrates and establishes substrate ordering.

### Mechanisms governing the temporal ordering of APC/C substrates in cell cycle

Ordered degradation of a vast array of substrates requires complex mechanisms that operate at multiple levels. It is now clear that APC/C is surprisingly plastic and can adopt different conformations during the cell cycle, and is regulated by a combination of the type of associated coactivator, substrate binding, inhibitory proteins, post-translational modifications of substrates and APC/C itself, to achieve this challenging feat.

#### Regulation of APC/C activity by coactivators

##### Conformational changes in APC/C induced by coactivators

Coactivators recognize specific targets, recruit them to APC/C, stimulate proper positioning of the catalytic core for ubiquitination, and recruit E2 enzymes [63–67]. Association of coactivators to the APC/C is regulated by phosphorylation of APC/C subunits.

**Fig. 2:**
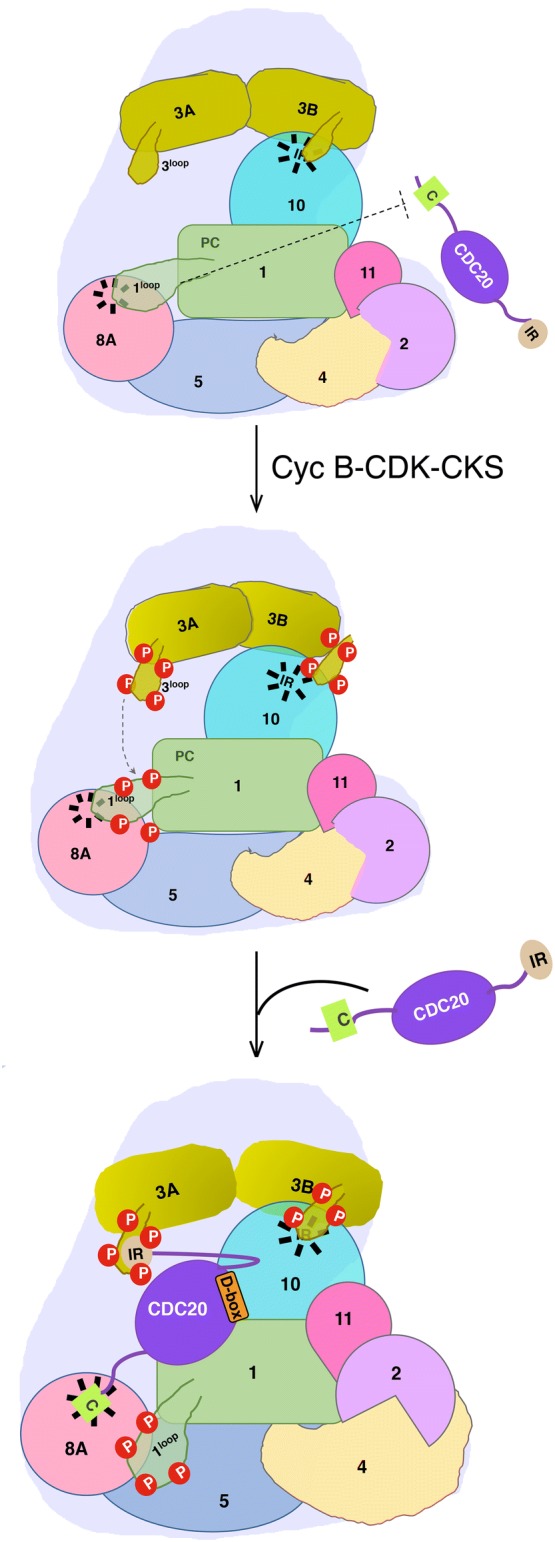
Activation of APC/C^CDC20^ by phosphorylation. In the unphosphorylated APC/C, the C-box binding site on APC8 is occupied by an auto-inhibitory loop of APC1, thus preventing the C-box of CDC20 to bind to APC/C. Phosphorylation of a loop in APC3 subunits results in recruitment of CDK1-CyclinB-CKS complex to this loop, leading to phosphorylation of the APC1 loop and displacing it from the C-box binding site, and allowing the CDC20 C-box to associate with APC8. The IR tail of CDC20 interacts with APC3A while the phosphorylated loop of APC3B interacts with the IR tail of APC10. Coactivator binding induces the movement of the catalytic core in the ‘up’ position

As discussed earlier, the coactivators are connected to the APC/C through several interactions via flexible linkers (Fig. 1). The interaction of the coactivators with APC/C is blocked in the interphase by a disordered loop from APC1 that occupies the C-box binding site of APC8/CDC23 (Fig. 2) [9]. Additionally, in the absence of the coactivator, a disordered loop in APC3/CDC27 blocks its IR-tail binding region. The block in APC/C is relieved upon phosphorylation of the serines in APC1 loop [9, 68, 69]. It has been suggested that coactivator engagement with the APC8/CDC23 via the C-box may result in conformational changes in the entire TPR lobe that can open the APC3 groove [11]. This allows the interaction of the IR-tails of Apc10, and coactivator CDH1, with APC3/CDC27. The binding of the coactivators allows repositioning of the platform and catalytic core in close proximity of the substrate-binding module (Fig. 2). This is aided by the release of the catalytic core from interaction with APC4 in Apo-APC/C. This, in turn, makes the APC2 C-terminal and APC11 more mobile to assume an upward position, thereby promoting the catalytic activity of the ligase (Fig. 2).

**Fig. 3:**
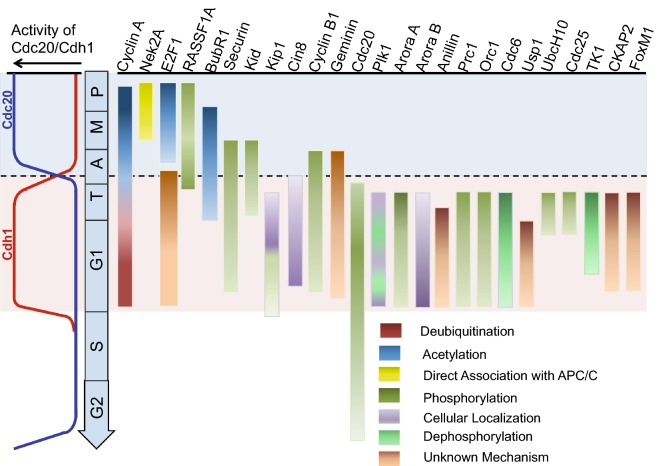
Post-translational modifications and the order of substrate degradation. Temporal pattern of APC/C activity in context with CDC20 (in blue) and with CDH1 (in red) in different cell cycle phases is shown on the left. Substrates are shown on top in the order of their degradation. The blue box denotes the CDC20-mediated, and orange box shows the CDH1 mediated degradation of the substrates. The substrates undergo various post-translational modifications that also contribute to the order in which they are degraded. These PTMs are color coded, with darker to lighter shading, where darker shade represents higher amount of protein and vice versa

While some substrates are recruited by both CDC20 and CDH1, others are specific to each coactivator. Therefore, cell cycle dependent degradation of some substrates may start during mitosis by APC/C^CDC20^ and continue with APC/C^CDH1^ till the late G1 phase (Fig. 3). The coactivators have to position the substrates for ubiquitination such that the UB carrying E2 enzyme, and substrate lysine are close together. This is achieved by the binding of the coactivators to the determinants in the substrates.

##### Destruction motifs recognized by the coactivators

The coactivators bind to the substrates by recognizing short linear motifs (SLiMs) called degrons. The best studied SLiMs are a nine residue motif called the Destruction-box (D-box) [70], KEN-box (Lys-Glu-Asn) [63], ABBA motif [71], and CRY-box [72]. Several others, like, O-box [73], and GxEN-box [74], are non-canonical D- and KEN box motifs (Table 1). The degrons interact with the C-terminal β-propeller domain of the coactivators. While D-box binding site lies between the blades of the propeller, KEN-box binds to the top of the propeller. The diversity of such motifs indicates that not all such motifs have been identified till date.

**Table 1:** Motifs recognised by CDC20 and CDH1 in known APC/C substrates

Motif	Consesus sequence	Coactivator	Substrate	Function	References
D-box	Rx^a^xLx[D/E][Ø]xN[N/S]	CDC20, CDH1	Securin, Cyclins	Proteolytic degradation	[70]
KEN-box	KENxxxN	CDH1	CDC20, NEK2A	Proteolytic degradation	[145]
ABBAmotif	Fx[ILV]^c^[FHY]x[DE]	CDC20	Cyclin A, BUBR1, BUB1 and ACM1	Proteolytic degradation	[71]
A-box	QRILGPS*^b^NVPQRV	CDH1	Aurora A kinase	Regulatory domain for proteolytic degradation	[35]
O-box	AS[P]LT[E][K][N][A]K	CDH1	ORC1	Proteolytic degradation	[73]
CRY-box	CRYxPS	CDH1	CDC20	Proteolytic degradation	[72]
GxEN	GxEN	CDC20, CDH1	XKID (Xenopus chromokinesin Kid)	Proteolytic degradation	[74]
SPO13	QK[P]LQ[E][K][T][P]N	CDH1	SPO13	Proteolytic degradation	[146]
CIN8P	KM[P]LR[L][S][N][I]N	CDH1	CIN8P	Proteolytic degradation	[147]
TEK-box	R/KxxTxKT	CDH1	Securin	K-11 ubiquitination	[148]
C-box	DRYIPHR	CDH1, CDC20	APC	APC/C association	[64]

^a^x signifies any amino acid residue

^b^* indicates one member of a closely related family of amino acids

^c^[] square brackets indicates any of the bracketed amino acid

Multisite binding of these SLiMs influences the processivity, selectivity and timing of degradation of APC/C substrates [75]. These SLiMs are also present in APC/C inhibitory proteins, like Emi1, that bind in a manner similar to MCC and inhibit the recognition of D-box substrates, and binding of the E2s to APC/C. Binding of the SLiMs is not sufficient to fully activate APC/C, as shown by the requirement of the coactivators to stimulate the activity of APC/C, even if the substrates are fused directly to the complex [75]. We refer the readers to an excellent and exhaustive review on the diversity and evolution of degrons by Davey and Morgan [76].

D-box (RxxLx[D/E][Ø]xN[N/S], where ‘Ø’ is a hydrophobic, and ‘x’ is any amino acid) is an important interacting motif that is recognized by CDH1 and APC10 in a bipartite manner. C-terminal hydrophilic region of the degron interacts with C-terminal IR-tail of APC10/DOC1, promoting high affinity binding of N-terminal of the degron with CDH1 propeller [77]. D-box based interactions are suggested to play an important role in determining the extent of processivity of multi-ubiquitination of APC/C substrates, thereby ordering substrate degradation [14]. Single point mutation of any of the three conserved residues of the D-box of wild-type Securin converts it from being multiubiquitinated by APC/C^CDH1^ to monoubiquitinated, with a higher dissociation rate from the APC/C, while two point mutations result in slow rate of Securin ubiquitination, suggesting that the affinity of the substrate partly determines the processivity of substrate ubiquitination [14].

Multiple lysines in the neighborhood of degrons are targeted for ubiquitination by APC/C [78]. Therefore, it is not surprising that the degrons are usually present in the unstructured, flexible regions of the substrate proteins. While direct affinity measurements of APC/C substrates have not yet been done, it is believed that the dissociation constant of a degron is in micromolar range. Different degrons, if present in a substrate, are closely spaced, and reflect the spacing between the degron binding sites on the coactivators. Cooperativity between different degrons on the same target is likely to bring the dissociation constant down to low nanomolar range. This multisite binding is likely to contribute to substrate ordering as shown for Cyclin A and NEK2A that have multiple degrons, and are degraded early even if SAC is inactivated [33, 79]. The ABBA motif of Cyclin A and BUBR1 bind to the same site on CDC20, thus competing for CDC20 binding [71, 80].

Substrates with tighter binding affinity for APC/C would be expected to compete with lower binding affinity substrate and should get ubiquitinated earlier, if these substrates share the same pool of APC/C [79]. This is observed for successful competition by CDC13 to delay Securin degradation by APC/C^CDC20^ in *S. pombe* [11]. But the S-phase cyclin, CLB5, cannot compete with Securin in *S. cerevisiae* [79]. While, cooperative, multi-site interactions may make a substrate more competitive, not all substrates show strong degron cooperativity. For example, Securin has both a D-box and a KEN-box, but deletion of either motif still results in its efficient ubiquitination [79]. This suggests that other intrinsic mechanisms also contribute to substrate ubiquitination by APC/C. It is likely that parts of substrates, other than the degrons, interact with APC/C subunits, and modulate the overall affinity of the substrate, and possibly also the stability of active conformations of APC/C. Structural and biophysical studies of APC/C with different substrates will be crucial to understand such intrinsic properties of the substrates.

##### Modulation of degron affinity

The affinity of the degrons can be modulated by post-translational modifications, or interactions, close to, or within the degrons [81]. Additionally, many degrons, and sequences surrounding them, show divergence from the consensus sequence that is likely to affect the affinity and specificity for the coactivator [76]. The lysine residue in the KEN box is frequently ubiquitinated in vivo, and may alter substrate affinity to the APC/C, thereby regulating the timing of destruction [81]. Phosphorylation close to the D-box can either increase or decrease the affinity of the degron depending on the preferred residues around the core degrons, and effect on structural stability of the degron [33, 82, 83].

#### Post-translational modifications (PTMs)

Subunits of the APC complex and many of its substrates are known to be targeted by various PTMs. However, very little information is available about the functional outcome of each of these PTMs, and the possible cross-talks and competition between them. The best understood PTMs, and how they affect key processes, are phosphorylation and ubiquitination. These two PTMs act at multiple levels and are crucial for temporal ordering of the APC/C activity and substrates.

##### Regulation of APC/C activity by phosphorylation

Phosphorylation functions at several different levels to regulate the activity of APC/C and in the selection of substrates. In early mitosis, APC/C is activated by the phosphorylation of its core subunits. Several different kinases—CDK1, PLK1, and Protein kinase A (PKA) are involved in the phosphorylation of APC/C during mitosis [84–86]. Phosphorylation of APC/C results in a change in the activity and localization of APC/C [80, 87, 88]. In vitro experiments show that CDK1 and PLK1 mediated phosphorylation activates APC/C, but PKA inhibits the activity towards Cyclin B, even in the presence of activators [84]. Estimates of the number of phosphorylation sites have been made using mass-spectroscopy approaches in both yeast and human APC/C, that show 43 sites in the human APC/C [89–91]. Mutagenesis studies suggest that loss of any CDK1 consensus phosphorylation site in APC/C leads to defects in mitotic events [85]. The kinetics, regulation, and effects of phosphorylation of these different sites remains a challenge but some studies suggest that these may be ordered events (CDK1 followed by PLK1), as phosphorylation by CDK1 is proposed to create a docking site for PLK1 [3].

CDK1 phosphorylates both APC/C subunits, and CDC20. Phosphorylation of the autoinhibitory loop of APC1, located in proximity to C-box binding site and close to the contact site between APC1 and N-terminal domain (NTD) of CDC20, exposes sufficient CDC20 binding sites on APC/C (Fig. 2) [91, 92]. Both CDC20 and CDH1 have multiple phosphorylation sites. Previously, it was demonstrated that phosphorylation of APC/C subunits promotes binding of CDC20 to APC/C and activates the APC/C^CDC20^ complex [88, 93], while phosphorylation of CDH1 by Cdks inhibits its binding to APC/C core complex, thereby inactivating APC/C^CDH1^ from the late G1 phase to the mitotic exit [94]. Recent studies show the phosphorylation of CDC20 to be inhibitory for binding to APC/C. Since both CDC20 and APC/C are phosphorylated by CDK1, the association of CDC20 with APC/C in mitosis could not be explained till recently. It turns out that the high specificity of mitotic phosphatase PP2A for threonines, rather than serines solves this problem. While the CDK1 phosphorylates CDC20 on threonine residues, it phosphorylates APC/C subunits on serines [95, 96]. Thus, dephosphorylation of CDC20 by PP2A promotes the affinity of CDC20 to phosphorylated and activated APC/C. CDK1 also phosphorylates the conserved mammalian kinase PLK1 in vitro, resulting in synergistic phosphorylation of multiple subunits of APC/C [97]. Phosphorylation of Ser92 residue of CDC20 interferes with the recruitment of E2 enzyme UBE2S to the APC/C and impairs the catalytic activity of APC/C [98]. PP2A mediated dephosphorylation of Ser92 of CDC20 allows UBE2S to be recruited to APC/C and activating it.

The phosphorylation sites targeted by CDK1 in CDH1 are serines. Similar to CDC20, phosphorylated CDH1 does not interact with APC/C, but unlike CDC20, that can be dephosphorylated by PP2A, CDH1 can be dephosphorylated only after CDK1 activity goes down [99, 100]. The phosphorylation status of CDH1 also governs its subcellular localization. The difference in residue preference of phosphatases thus governs the temporal association of the two coactivators to APC/C, thereby ensuring ordered substrate degradation.

Phosphorylation can also inhibit APC/C^CDC20^ activity. During interphase, nuclear Cyclin A–CDK2 phosphorylates the CDC20 at the inhibitory sites close to C-box, without affecting its binding with APC/C. This reduces the interphase APC/C^CDC20^ activity compared to that observed during mitosis [101–103]. The negative regulation of interphase APC/C^CDC20^ activity by Cyclin A/CDK2 allows accumulation of mitotic cyclins and ensures efficient mitotic entry [104].

##### Regulation of APC/C substrates by phosphorylation

Phosphorylation status of some substrates also determines their recognition by the APC/C and influences the precise timing of their degradation. Phosphorylation of specific residues can result in changes in the substrate conformation that either expose or occlude the degron availability. Phosphorylation sites are often found in or near the D-box of APC/C substrate, and might be involved in controlling the degradation time [105]. Phosphorylation of the acidic residue at position + 6 of the D-box generally promotes ubiquitination, while phosphorylation of the basic residue at position + 2 of the D-box stabilizes the substrate [8]. Similarly, mitotic phosphorylation of Aurora kinase A at Ser53 of the A-box motif inhibits its ubiquitination, and dephosphorylation of Ser53 during mitotic exit stimulates its ubiquitination. This is due to a conformational change that makes its D-box accessible to APC/C^CDH1^ leading to its timely destruction [35]. Conversely, phosphorylation of CDC6 prevents its recognition by APC/C^CDH1^ [106], and phosphorylation of SKP2 by AKT [107] impairs its APC/C^CDH1^ mediated degradation. Phosphorylation of two CDK1 sites near the D- and KEN-box of Securin enhances its ubiquitination in vitro [105, 108].

During mitotic exit, most substrates are dephosphorylated for G1 to be established. It is possible that substrates are dephosphorylated in an ordered manner. Ordered dephosphorylation of yeast CDK1 substrates, and of CDH1 in late mitosis could be made possible by a moderate change in the ratio of the CDC14 phosphatase to CDK1 kinase during mitotic exit. This change may be detected by CDK1 substrates and CDH1, that may then get dephosphorylated at discrete thresholds [109]. Interestingly, after APC/C^CDC20^ dependent degradation of Securin, inhibitory phosphorylation of Separase by CDK1 creates phosphor-sites on Separase for stable binding of CDK1, thereby taking it away from APC/C. Therefore, Separase acts as an inhibitor of Securin at metaphase, and then of CDK1 in late anaphase, assisting APC/C in mitotic exit [110]. Thus, phosphorylation and dephosphorylation of CDK1 substrates may act as key factors that determine the sequential degradation of APC/C substrates.

Phosphorylation of APC/C subunits is, however, not sufficient for destruction of Securin and Cyclin B, as ubiquitination of these substrates is inhibited by SAC until metaphase [111]. On the other hand, degradation of Cyclin A and NEK2A occurs even when the SAC is active. CDC20 can associate with Cyclin A even before cells enter mitosis [112]. Other than multiple degrons that facilitate this, Cyclin A forms a trimeric complex with its partner CDK and CKS protein (Fig. 5). The CKS1 protein has a phosphate-binding site composed of conserved positively charged residues and can bind to activated phosphorylated APC/C with high affinity [113, 114].

To exit the mitosis, CDC20 switches from being a coactivator to become a substrate of APC/C ^CDH1^. This is due to the phosphorylation of Ser170 in the CRY-box of CDC20 by PLK1, that induces the binding of CDC20 to active APC/C^CDH1^ for degradation [115].

##### Ubiquitination/deubiquitination of substrates

**Fig. 4:**
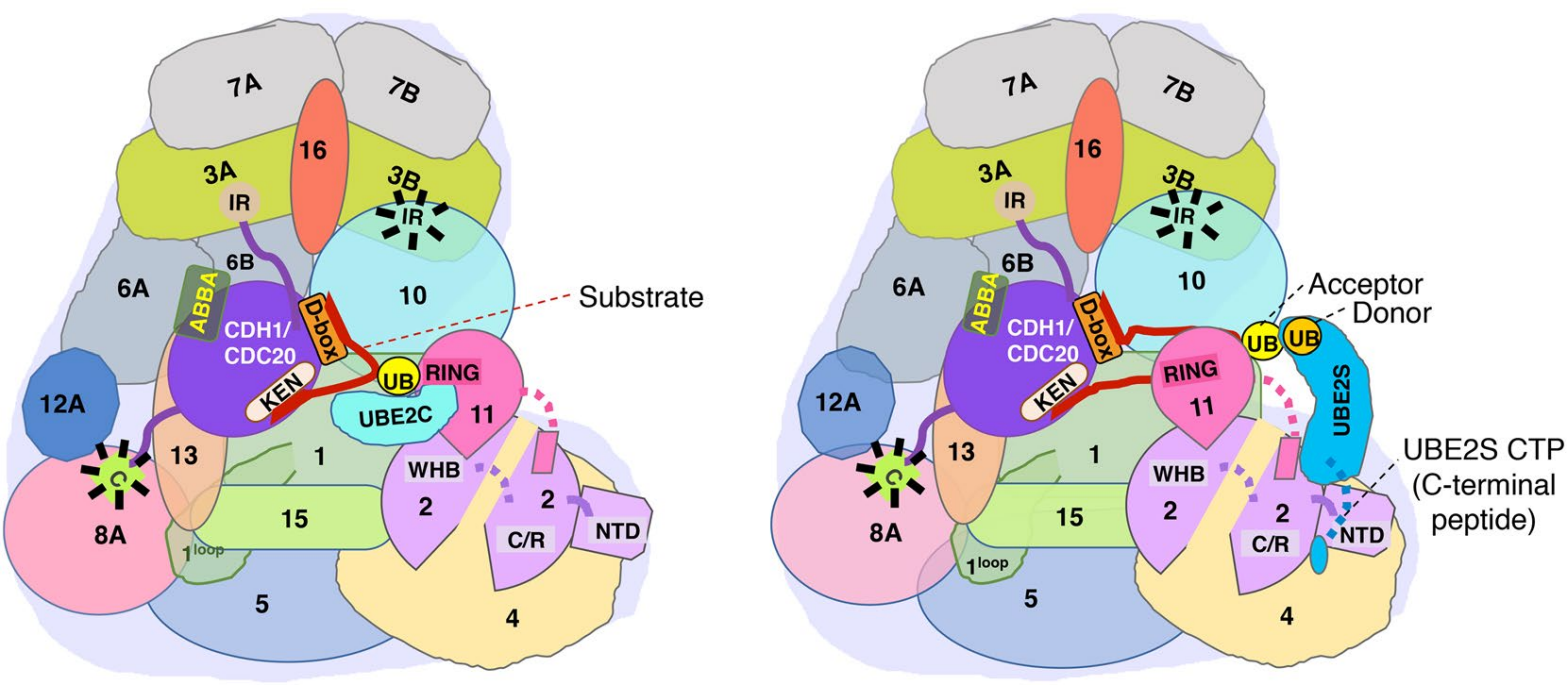
Different modes of binding of UBE2C and UBE2S to APC/C and effects on ubiquitination of the substrates. Upon engagement of the coactivator (purple) bound to the substrate (shown as a solid red line, with D- and KEN-boxes), UBE2C interacts with APC11 and the WHB domain of APC2 interacts with the backside of UBE2C. This arrangement restricts the sample space that can be explored by UBE2C and allows only a few ubiquitin molecules to be attached to the substrates. UBE2S interacts with a different region of APC11 that is away from the RING domain, while the C-terminal peptide of UBE2S binds to a site between APC2 and APC4 via a flexible linker. The RING domain of APC11 interacts with the acceptor ubiquitin (yellow) on the substrate and presents its K11 residue for accepting a ubiquitin (orange) from UBE2S. Flexible linkers of APC11 and UBE2S are shown by dashed lines

APC/C cooperates with a pair of E2s, i.e., an ‘initiator E2’ UBE2C/UBCH10, and an ‘elongator E2’ UBE2S. While UBE2C/UBCH10 adds multiple mono-ubiquitins or short Ub chains on the substrates, UBE2S adds K-11 linked polyubiquitin chains on UBE2C/UBCH10 modified substrates. This is achieved by the distinct ways in which these E2s interact with APC/C (Fig. 4). Coactivator binding changes the conformation of the cullin-RING catalytic module of APC/C from ‘down’ to ‘up’ position resulting in a clamp-like engagement of UBE2C/UBCH10 due to interaction with APC11 RING domain, and through backside binding to APC2 WHB domain [116]. This confines and positions its active site towards the substrate, and may be the reason why this E2 functions as an ‘initiator’ E2. UBE2C/UBCH10 is released from APC2 WHB interaction for charging by E1 for another monoubiquitination. UBE2S, on the other hand, is recruited to distinctive surfaces on APC/C to extend K-11 linked polyUB chains on the acceptor UB-primed substrate (Fig. 4). The C-terminal peptide like extension (CTP) of UBE2S buries between APC2 and APC4 two helix bundle to interact with APC/C platform in a RING independent manner. The catalytic domain of UBE2S interacts with APC2, while APC11 RING domain engages with the substrate-linked UB acceptor to enhance its interaction with UBE2S active site [117]. This interaction facilitates reloading of UB onto APC-bound UBE2S to enhance processive polyubiquitination of substrates with K-11 linked chains. Whether a substrate can be modified by both UBE2C/UBCH10 and UBE2S at the same time is not yet known.

The difference in the processivity of ubiquitination has been proposed to play an essential role in temporal substrate ordering [14]. While the action of these two E2s result in processive and distributive ubiquitination of substrates, both extrinsic and intrinsic features of the substrates determine the residence time with APC/C. As mentioned above, direct affinity of various degrons has not been measured, but a range of affinities is likely to be present due to differences from the canonical motifs. Moreover, single-molecule studies show that UB modification increases the residence time of the substrate on APC/C, thus increasing the likelihood of processive ubiquitination [82]. This is known as ‘processive affinity amplification’ that allows a range of stabilities of the substrates.

Processivity can also be increased by the multimerization of the APC/C. Support for this model is provided by the observations that yeast APC/C dimer having four catalytic sites is twofold more active, and sevenfold more processive than monomeric APC/C [118]. If human APC/C dimer also exists, then possibly less stable APC/C dimer would be ubiquitinating processive substrates, while APC/C monomers may ubiquitinate distributive substrates.

Additionally, substrates can be ubiquitinated by two distinct mechanisms: either in cis, i.e., substrate leading to its autoubiquitination; or in trans, in which one molecule of APC/C bound substrate acts as a coactivator, ubiquitinating the other molecule of free substrate. Decline in CDC20 levels after depletion of its substrates can be explained by *cis*-autoubiquitination, whereas in early mitosis, presence of its substrates blocks its cis-autoubiquitination [119]. The degradation of CDC20 switches to the trans-ubiquitination mode once APC/C^CDH1^ is activated.

While the rate of ubiquitination sets the timing for the initiation of degradation, proofreading mechanisms, like deubiquitination, can also delay the degradation of some substrates and contribute to the correct timing of APC/C substrate degradation. Kinetic proofreading of multiubiquitinated APC/C substrates can be a striking feature for establishing ordering of substrates destruction [79]. This is exemplified by competing ubiquitination and deubiquitination of MCC associated CDC20 (CDC20^MCC^) which is important for shutting down, or sustaining the SAC. UBE2C/UBCH10 ubiquitinates CDC20^MCC^ which results in disassembly of SAC, while USP44 mediated deubiquitination of CDC20^MCC^ sustains it [120, 121]. Moreover, a deubiquitinating enzyme, OTUD7B/Cezanne, specifically targets K-11 linked chains assembled by APC/C on its substrates, in a cell cycle regulated manner [122]. This allows stabilization of mitotic substrates and regulated progression of mitosis. Thus, dynamic antagonistic effect of ubiquitination and deubiquitination generates a switch like transition from metaphase to anaphase by regulating CDC20^MCC^ by UBE2C/UBCH10, and substrate stability by OTUD7B/Cezanne. Further, UBE2C/UBCH10, which is considered to be a distributive substrate of the APC/C, is prone to deubiquitination and is degraded late in G1 phase.

##### Acetylation

Acetylation has also emerged as an additional control of cell-cycle progression by modifying the substrates for timely degradation. Acetyl transferase p300/CBP-associated factor (PCAF) transfers acetyl groups to ε-amino group of the specific lysine residues of few substrates, and has been proposed to have intrinsic ubiquitin activating/conjugating and ligase activities [123, 124]. PCAF associates with Cyclin A and acetylates it at four specific lysines located in the N-terminal domain of Cyclin A. This targets Cyclin A for degradation in early mitosis, regardless of SAC [125]. Perhaps, acetylation helps in the correct attachment of the UB molecules on specific sites for SAC independent Cyclin A degradation. Similarly, PCAF acetylates BUBR1 at K250 in prometaphase and acetylated BUBR1 binds to CDC20 when SAC is active [126]. When SAC is switched off, BUBR1 gets deacetylated, which promotes its ubiquitination, thus disassembling the MCC. Thus, BUBR1 acetylation/deacetylation status provides a new mechanism of regulating APC/C activity and mitosis exit, and serves as a molecular switch to convert BUBR1 from an APC/C inhibitor, to a substrate of APC/C complex (with CDC20 in mitosis, and with CDH1 after mitosis exit) [126]. Moreover, it is reported that acetylation and phosphorylation of BUBR1 is coordinated in cells [127]. Details of how this coordination is achieved is currently not known.

#### Regulation of APC/C by the spindle assembly checkpoint (SAC)

**Fig. 5:**
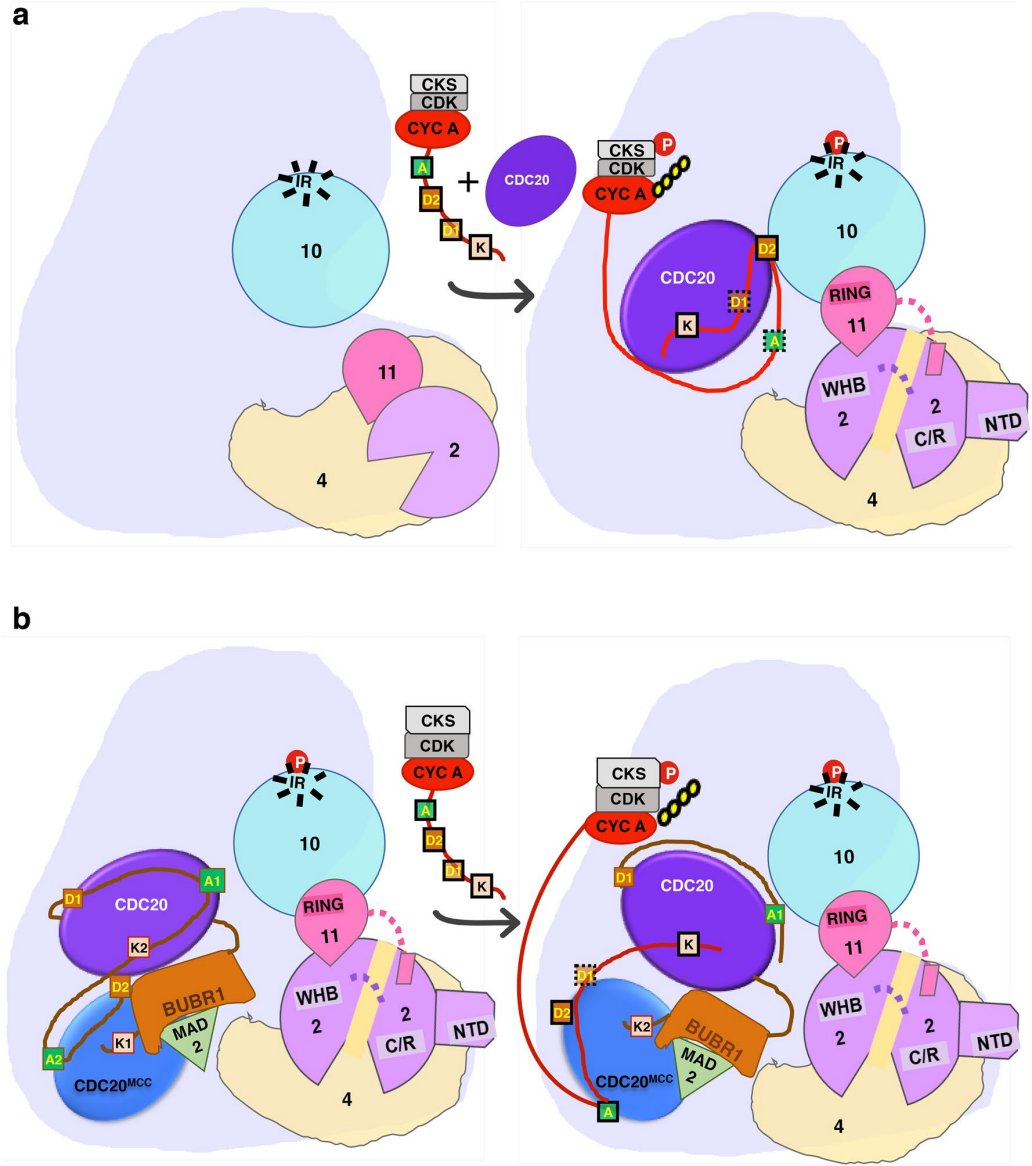
Spindle assembly checkpoint (SAC) independent ubiquitination of Cyclin A2. **a** Phosphorylation of inactive, apo-APC/C leads to binding of CDK1-Cyclin A2-CKS1 complex to CDC20. The degrons of Cyclin A2 can bind in two different modes to APC/C^CDC20^, only one mode is shown here that engages KEN- (K) and non-canonical D-box (D2), and activates ubiquitination of Cyclin A2. **b** Cyclin A can also bind to APC/C^CDC20^-MCC complex. In the closed APC/C–MCC conformation, BUBR1 forms a lariat like structure between APC/C^CDC20^ and CDC20^MCC^ via its multiple degrons. ABBA-box of Cyclin A2 (A) competes with the ABBA-box 2 of BUBR1 (A2) and can bridge both CDC20 molecules in APC/C by interaction of its D2- and KEN- boxes. This is proposed to induce the open conformation of APC/C–MCC and facilitates Cyclin A2 ubiquitination. For clarity, only relevant subunits are shown here. Yellow circle shows ubiquitination, degrons bounds with dashed lines indicate no interaction, those with solid lines indicate binding. Flexible linkers are shown by dashed lines. P denotes phosphorylation

Surveillance mechanisms by the SAC prevent the late events until early events, like proper kinetochores attachment, are completed. SAC creates a boundary that prevents the premature degradation of APC/C^CDC20^ substrates like Cyclin B and Securin, thus contributing to substrate ordering. SAC component proteins Mitotic arrest deficient 2 (MAD2), BUBR1 (MAD3), and Budding uninihibited by bezimidazole (BUB3) form an inhibitory MCC in which MAD2 and BUBR1 interact directly with CDC20 [114, 128–132]. BUBR1 has two copies of both D-box (D1, D2) and KEN-box (K1, K2), and three copies of ABBA box (A1–A3). Except A3, other six motifs intertwine to form a lariat-like structure between both APC/C^CDC20^ and CDC20^MCC^, and block the degron dependent interaction of its authentic substrates to both coactivator subunits (Fig. 5) [8]. Through TPR domain of BUBR1, MCC contacts APC2^WHB^. This obstructs the UBCH10 binding to APC/C catalytic core, thereby inhibiting ubiquitination catalysis [133]. The APC/C–MCC can adopt two conformations: closed and open. These conformations are influenced by the order-to-disorder transitions of APC15 subunit. In the closed state, MCC blocks the UBE2C binding site on the catalytic module, whereas in the open state, rotation of MCC away from the catalytic module allows UBE2C to bind to APC/C. The open conformation allows autoubiquitination of CDC20^MCC^ (Fig. 5) and dismantling of MCC. Thus, the open and closed states of MCC regulate the reciprocal regulation between APC/C^CDC20^ and APC/C bound MCC. Kinetochore attachment activates the p31^comet^ protein, which antagonizes SAC by removing MAD2 from MCC. It also prevents assembly of new MCC by competing with BUBR1 for binding to c-MAD2 [134].

Relative time difference between degradation of Cyclin A and Securin is important to contribute to ordered progression of mitosis. While Cyclin A promotes the detachment of kinetochores from the microtubules, its destruction stabilizes the kinetochore-microtubule attachments [135]. Thus, Cyclin A degradation before mitosis is critical for correct chromosomal segregation. Based on information obtained from biochemical and structural studies, a new model has been proposed to explain the SAC resistant degradation of Cyclin A [136]. The paper by Zhang et al. shows that besides a canonical D-box, Cyclin A2 also contains a non-canonical D-box (D2 box). Cyclin A2 can engage with APC/C in two different ways: either through a cooperative binding via the canonical D-, KEN- and ABBA boxes, or via the D2- and KEN-boxes. These two modes of binding may be responsible for different efficiency of Cyclin A2 ubiquitination, possibly due to different conformations of the WD40 propeller of CDC20. Further, CDK-Cyclin A2-CKS1 complex can stably interact with APC/C–MCC complex by competitive displacement of the ABBA box of BUBR1 by the ABBA box of Cyclin A2. Finally, the D2 box of Cyclin A2 interacts with CDC20^MCC^, and its KEN- and ABBA-boxes bridge the CDC20 associated with APC/C and with MCC. This induces the open form of APC/C–MCC that can engage with the E2 and enables ubiquitination of Cyclin A2 (Fig. 5). This is a very attractive model and it is possible that similar to Cyclin A, SAC-inhibited APC/C^CDC20^ may recognize distinct features on other checkpoint independent substrates.

#### Subcellular localization and substrate competition

Other than the mechanisms discussed above, localization and partitioning of APC/C and its substrates in different cellular compartments, are also likely to contribute to substrate ordering to some extent. For example, Securin is present mostly in phosphorylated form in the cytoplasm. Only a small fraction of total Securin pool is in the nucleus that binds and inhibits Separase on the chromosomes [137]. Fully activated APC/C^CDC20^ first targets the bulk of free phosphorylated Securin and then the small pool of Separase bound Securin on the chromosomes.

Increased availability of active APC/C might promote the substrate ubiquitination in a compartment specific manner. There is evidence to indicate that spindle pole associated APC/C pool is specifically inactivated [138], whereas APC/C pool associated with chromosomes is much more active compared to the cytoplasmic APC/C pool [139]. Thus, APC/C substrates that promote the formation of spindle are protected from ubiquitination, while being concentrated on mitotic spindle microtubules [140]. These substrates are differentially localized or post-translationally modified for their timely degradation. Synergistic phosphorylation of Cyclin B1 by MAPK (ERK2) and PLK1 promotes rapid nuclear translocation of Cyclin B1 at G2/M phase, and the active pool of APC/C, that is associated with chromosomes, leads to ubiquitination of Cyclin B1 [141]. The spindle and kinetochore-associated (SKA) complex has been shown to enhance the binding of APC/C to chromosomes, but the detailed mechanism of how this is achieved is currently not understood [139].

Given the functional importance of the coactivators in the temporal regulation of substrate degradation, it is interesting that several alternatively spliced transcript variants of human CDH1 have been reported in the genome database. One shorter form of CDH1 lacks the beta-propeller blade that participates in binding to the D-box. It also has fewer phosphorylation sites, and no nuclear localization signal compared to the full length isoform [142], but its functional and biological significance is not known at present. Does it allow APC/C to discriminate between substrates by having a different substrate preference, or, it has a cell cycle independent function, is not known. Some of the non-cell cycle substrates of APC/C are cytosolic, e.g., PFKFB3 and Glutaminase 1, but their degradation is coordinated with cell proliferation [64, 65]. It is a possibility that APC/C associated with the shorter isoform of CDH1 regulates degradation of these cytoplasmic substrates. More precise localization of various pools of APC/C and its substrates may provide further insight into how APC/C targets its various susbtrates in different subcellular locations.

## Future perspectives

APC/C has emerged as a central control knob of the cell cycle, regulating transition from one phase to another phase as well as regulating neuronal development, myogenic differentiation, apoptosis and metabolism. Temporal regulation of substrate ubiquitination by APC/C is critical for the proper timing of cell cycle events and possibly for coordinated regulation of other cellular processes with the cell cycle. Given its importance in cell cycle regulation, APC/C is considered a good candidate for cancer and anti-viral drug development. An understanding of the mechanism of substrate selection, binding and processivity of APC/C towards various substrates is a prerequisite to address cancer related issues, and developmental and viral diseases.

While the conformational dynamics due to binding of different partners has illustrated many aspects of substrate ordering by APC/C, many questions still remain. Quantitative and structural data with different substrates is needed to fully understand the substrate selection by this complex and how it computes different mechanistic controls to decide the fate of the substrate. Recent reports of APC/C independent inhibition of signaling protein SRC by CDH1 and inhibition of CDH1 function by an overactive SRC indicate a previously unknown mechanism of APC/C regulation by SRC signaling [143]. It is possible that there may be other such mechanisms that can regulate a complex and dynamic cancer network, and suggest additional layers of APC/C regulation that need to be explored to understand the tumor suppressor function of CDH1.

APC/C and its regulatory proteins are subjected to many post-translational modifications besides phosphorylation. APC/C subunits also undergo sumoylation and methylation. BUBR1 is sumoylated besides getting phosphorylated, acetylated and ubiquitinated. It is clear from the example of BUBR1 that some of these PTMs are crucial for a proper cell cycle and studies centered on how the PTM code is coordinated would be important to further our understanding of this complex ubiquitin ligase.

Current models to explain the substrate ordering by the APC/C assume a homogenous population of the complex to be present in the cell. However, different APC/C subpopulations are likely to exist in the cells. For example, APC/C can associate with pRB via CDH1 and promote SKP2 degradation. Similarly, TGFβ promotes degradation of SKP2 and CKS1 by APC/C^CDH1^ [144]. It is an open question whether TGFβ and pRB interact with other subunits of APC/C^CDH1^ and influence the orientation of the platform, and the flexibility and position of the catalytic core; or they increase the affinity of the SKP2 with CDH1 to allow for progressive affinity amplification? Additionally, the presence of the shorter isoform of CDH1 that can associate with core APC/C and reside in the cytosol opens up many questions, and suggests further complex controls that APC/C may be subjected to, in order to coordinate its various functions. It is likely that the cellular APC/C pool is more diverse than previously thought. This diversity in APC/C may contribute towards substrate ordering and the current models of substrate ordering by APC/C may need to be revisited once we learn more about these diverse interactions and subpopulations of APC/C and their functions.

## Data Availability

Not applicable.

## Abbreviations

AMA1: activator of meiotic APC1
APC/C: anaphase-promoting complex/cyclosome
BUB3: budding unihibited by benzimidazole 3
CAK: CDK activating kinase
CDK: cyclin dependent kinase
CKI: cyclin-dependent kinase inhibitor
CRL: cullin-RING ligase
CTP: C-terminal peptide
D-box: destruction box
E1: ubiquitin activating enzyme
E2: ubiquitin conjugating enzyme
E3: ubiquitin ligase
FZR1: fizzy-related 1
GLS1: glutaminase 1
ID2: inhibitor of differentiation 2
IR: isoleucine-arginine
KEN: lysine-glutamine-asparagine
MAD: mitotic arrest deficient
MCC: mitotic checkpoint complex
MFR1: meiotic fizzy related 1
NSC: neural stem cells
NTD: N-terminal domain
PC: Proteasome-Cyclosome
PCAF: p300/CBP associated factor
PFKFB3: 6-phosphofructo-2-kinase/fructose 2,6-bisphosphatase isoform 3
PKA: protein kinase A
PLK1: polo-like kinase 1
PTM: post-translational modification
RASSF1: Ras-association domain containing family 1
SAC: spindle assembly checkpoint
SCF: SKP-Cullin-F-box containing complex
SKA: spindle and kinetochore associated
SKP: S-phase kinase-associated protein
SliMs: short linear motifs
TPR: tetratricopeptide
UB: ubiquitin
WHB: winged helix B
XKID: Xenopus chromokinesin kid
ZBR: zinc-binding region
